# A decrease in anaerobic bacteria promotes *Candida glabrata* overgrowth while β-glucan treatment restores the gut microbiota and attenuates colitis

**DOI:** 10.1186/s13099-018-0277-2

**Published:** 2018-12-03

**Authors:** Rogatien Charlet, Clovis Bortolus, Melissandre Barbet, Boualem Sendid, Samir Jawhara

**Affiliations:** 1 U995/Team2, INSERM, 59000 Lille, France; 2 LIRIC-INSERM U995/2, Lille Inflammation Research International Center, University Lille, 1 Place Verdun, 59000 Lille, France; 3 Service de Parasitologie Mycologie, Pôle de Biologie Pathologie Génétique, CHU Lille, 59000 Lille, France

**Keywords:** β-Glucans, *Candida glabrata*, Microbiota, Colitis, Anaerobic bacteria, *Escherichia coli*, *Enterococcus faecalis*

## Abstract

**Background:**

The intestinal microbiota plays a crucial role in the maintenance of gut homeostasis. Changes in crosstalk between the intestinal epithelial cells, immune cells and the microbiota are critically involved in the development of inflammatory bowel disease. In the experimental mouse model, the development of colitis induced by dextran sulfate sodium (DSS) promotes overgrowth of the opportunistic yeast pathogen *Candida glabrata*. Conversely, fungal colonization aggravates inflammatory parameters. In the present study, we explored the effect of *C. glabrata* colonization on the diversity of the gut microbiota in a DSS-induced colitis model, and determined the impact of soluble β-glucans on *C. glabrata*-host interactions.

**Results:**

Mice were administered a single inoculum of *C. glabrata* and were exposed to DSS treatment for 2 weeks in order to induce acute colitis. For β-glucan treatment, mice were administered with soluble β-glucans purified from *C. glabrata* (3 mg per mouse), orally and daily, for 5 days, starting on day 1. The number of *C. glabrata* colonies and changes in microbiota diversity were assessed in freshly collected stool samples from each tagged mouse, using traditional culture methods based on agar plates. An increase in *Escherichia coli* and *Enterococcus faecalis* populations and a reduction in *Lactobacillus johnsonii* and *Bacteroides thetaiotaomicron* were observed during colitis development. This decrease in *L. johnsonii* was significantly accentuated by *C. glabrata* overgrowth. Oral administration of β-glucans to mice decreased the overgrowth of aerobic bacteria and IL-1β expression while *L. johnsonii* and *B. thetaiotaomicron* populations increased significantly. β-glucan treatment increased IL-10 production via PPARγ sensing, promoting the attenuation of colitis and *C. glabrata* elimination.

**Conclusions:**

This study shows that the colonic inflammation alters the microbial balance, while β-glucan treatment increases the anaerobic bacteria and promotes colitis attenuation and *C. glabrata* elimination.

## Background

The commensal intestinal microbiota helps to maintain stability and prevent overgrowth or infection with pathogenic bacteria. Gut microbiota dysbiosis has been associated with inflammatory bowel diseases (IBDs) [1]. IBDs are chronic inflammatory diseases of the gastro-intestinal (GI) tract, and include Crohn’s disease (CD) and ulcerative colitis (UC) [2]. Disruption of the gut microbiota as well as impairment of host immunity give rise to perturbations promoting the overgrowth of opportunistic yeast species such as *Candida albicans,* leading to increased yeast translocation and susceptibility to systemic infection [3–5]. Like *C. albicans*, *C. glabrata* can cause infections ranging from mucosal lesions to bloodstream or invasive infections in patients with altered immune or physiological responses [6]. The cell wall of *C. glabrata* plays a crucial role in the interaction of the yeast with its host. The yeast cell wall mainly contains of carbohydrate polymers linked with glycoproteins and phospholipids [7]. The three major components are β-glucans (polymers of glucose), chitin (a polymer of *N*-acetylglucosamine), and mannans [7]. β-1,3-glucans provide the major structural scaffold of the yeast cell wall and have different amounts of β-1,6 branches.

Different studies have demonstrated the biological properties of β-glucans, particularly their anti-inflammatory, antioxidant, and anti-tumor effects [8–10]. β-glucans can directly activate leukocytes, and further stimulate their phagocytic, cytotoxic, and antimicrobial activities. β-glucans are recognized by several receptors, in particular integrin α_M_β_2_, dectin-1 and TLR-4, thereby triggering an immune response [11–13].

In the present study, we studied the effect of β-glucans on the biodiversity of the gastrointestinal tract microbiota in a dextran sulfate sodium (DSS)-induced colitis model. We also analyzed the effect of β-glucans on intestinal inflammation and elimination of *C. glabrata* from the gut.

## Results

The impact of *C. glabrata* colonization on intestinal inflammation and gut microbiota changes was determined in a DSS induced-colitis model. The effect of oral administration of β-glucan on intestinal inflammation, the gut microbiota, and *C. glabrata* elimination was also investigated.

### Determination of inflammatory parameters

**Fig. 1:**
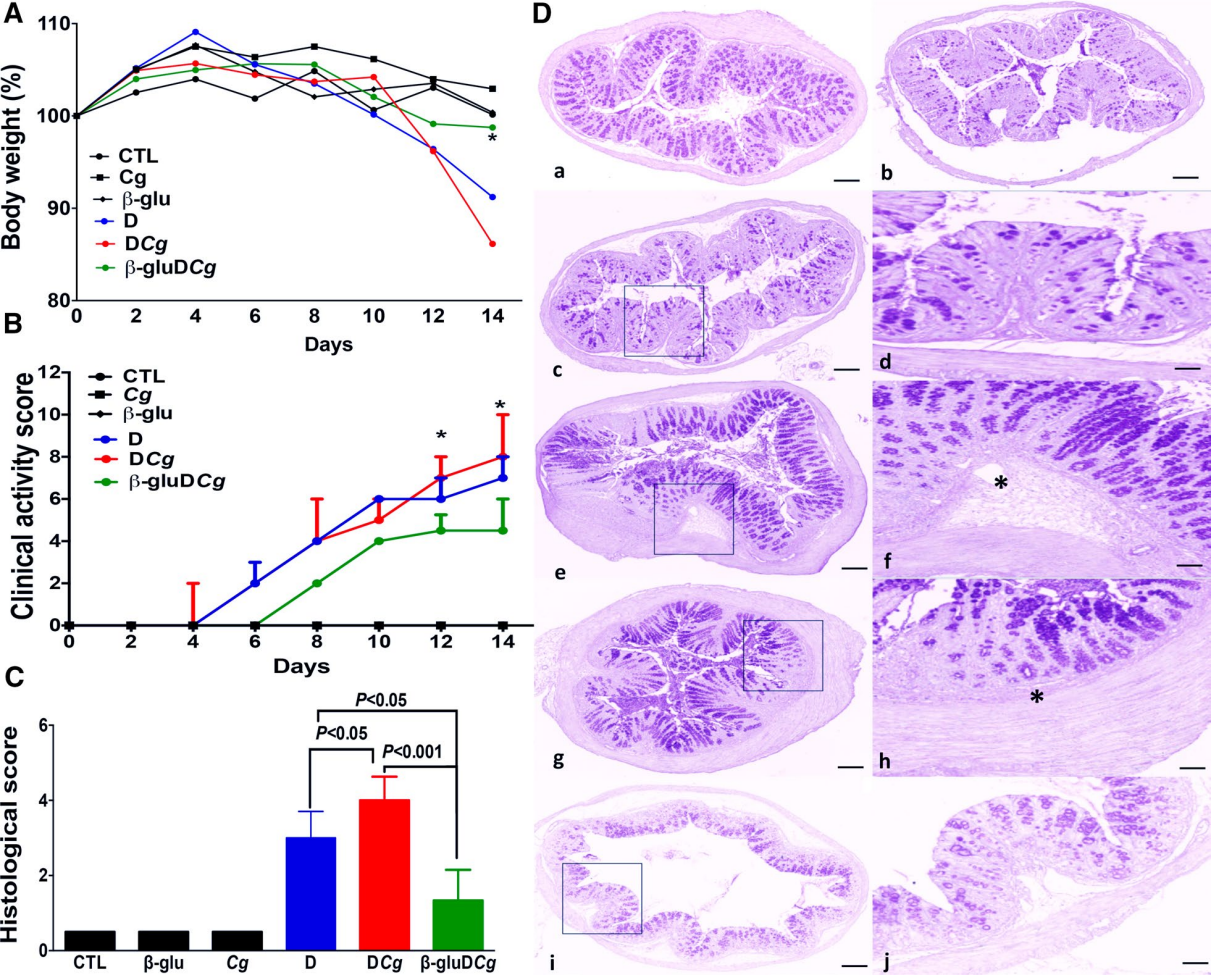
Effect of *C. glabrata* colonization on DSS-induced colitis. **A**, **B** Body weight and clinical analysis of DSS-induced colitis in mice. The six groups consisted of controls (CTL, water), *C. glabrata* alone (Cg), β-glucans (β-glu), DSS alone (**D**), DSS + *C. glabrata* (DCg), and β-glucans + DSS + *C. glabrata* (β-gluDCg). Clinical score was determined by analyzing changes in stool consistency, body weight loss, and presence of bleeding. The CTL, Cg, and β-glu groups did not show any signs of inflammation. Thus, the curves for CTL, Cg, and β-glu overlapped indicating the absence of clinical activity score for these three groups. **P < 0.001 for DSS (**D**) and *C. glabrata*-DSS (DCg) mice vs. controls (CTL), *C. glabrata* (Cg), β-glucans (β-glu) and *C. glabrata* + DSS + β-glucans (DCgβ-glu) groups. **C** Histologic scores. Mice were exposed to 2% DSS in drinking water for 14 days. Scores range from 0 (no changes) to 6 (extensive cell infiltration and tissue damage). **D** Histologic analysis of the colon in *C. glabrata* and DSS-induced colitis. Panel (a) is the control group (CTL) receiving only water. Panel (b) corresponds to colon sections from WT mice receiving *C. glabrata* only. Panel (c) corresponds to colon sections from mice receiving β-glucans only. Panel (e) correspond to colon sections from mice receiving DSS. Panel (g) corresponds to colon sections from mice receiving *C. glabrata* + DSS. Panel (i) corresponds to colon sections from mice receiving *C. glabrata* + DSS + β-glucans. In the absence of DSS, no inflammation signs in colon sections were observed between control groups (not inoculated) and those that received *C. glabrata* or β-glucans (panels a, b, and c). Panels (e) and (g) show a high inflammatory cell infiltrate in the colon wall structures and massive tissue destruction (asterisks). The scale bars represent 50 µm (a, b, c, e, g, and i) and 20 µm (d, f, h, and j)

Mice were exposed to 2% DSS in drinking water for 2 weeks to induce colitis. Mice developed colitis with inflammatory clinical signs of diarrhea, gross rectal bleeding, and body weight loss, starting from day 6 after DSS treatment (Fig. 1). Mice given water (control), *C. glabrata* only, or β-glucans showed no clinical signs of inflammation. In contrast, mice given DSS or *C. glabrata*-DSS showed body weight loss and an increase in the clinical score for inflammation. β-glucan treatment significantly reduced the clinical score for inflammation in *C. glabrata*-DSS mice (Fig. 1b).

The histologic score was significantly higher in *C. glabrata*-DSS mice than in DSS-treated mice (Fig. 1c). In contrast, β-glucan treatment significantly reduced the clinical and histologic scores of inflammation in *C. glabrata*-DSS mice. High leukocyte infiltrates, cryptic abscesses, and loss of epithelium occurring throughout the colon mucosa and submucosa were more frequently observed in the colons of *C. glabrata*-DSS mice than in mice treated with DSS. β-glucan administration reduced leukocyte infiltrates, epithelial damage and mucosal abscesses (Fig.1d).

### *C. glabrata* colonization and modification of the gut microbiota during colitis development

**Fig. 2:**
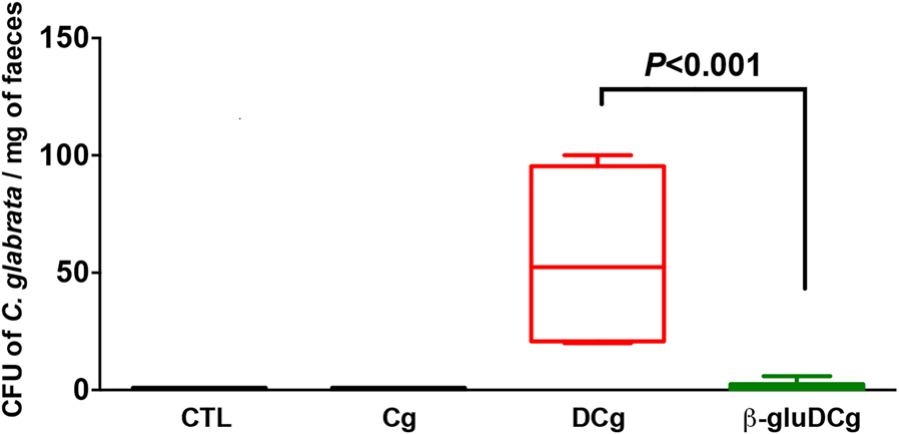
*C. glabrata* colonization in mouse DSS-induced colitis. Number of *C. glabrata* colony forming units (CFU) recovered from stools

**Fig. 3:**
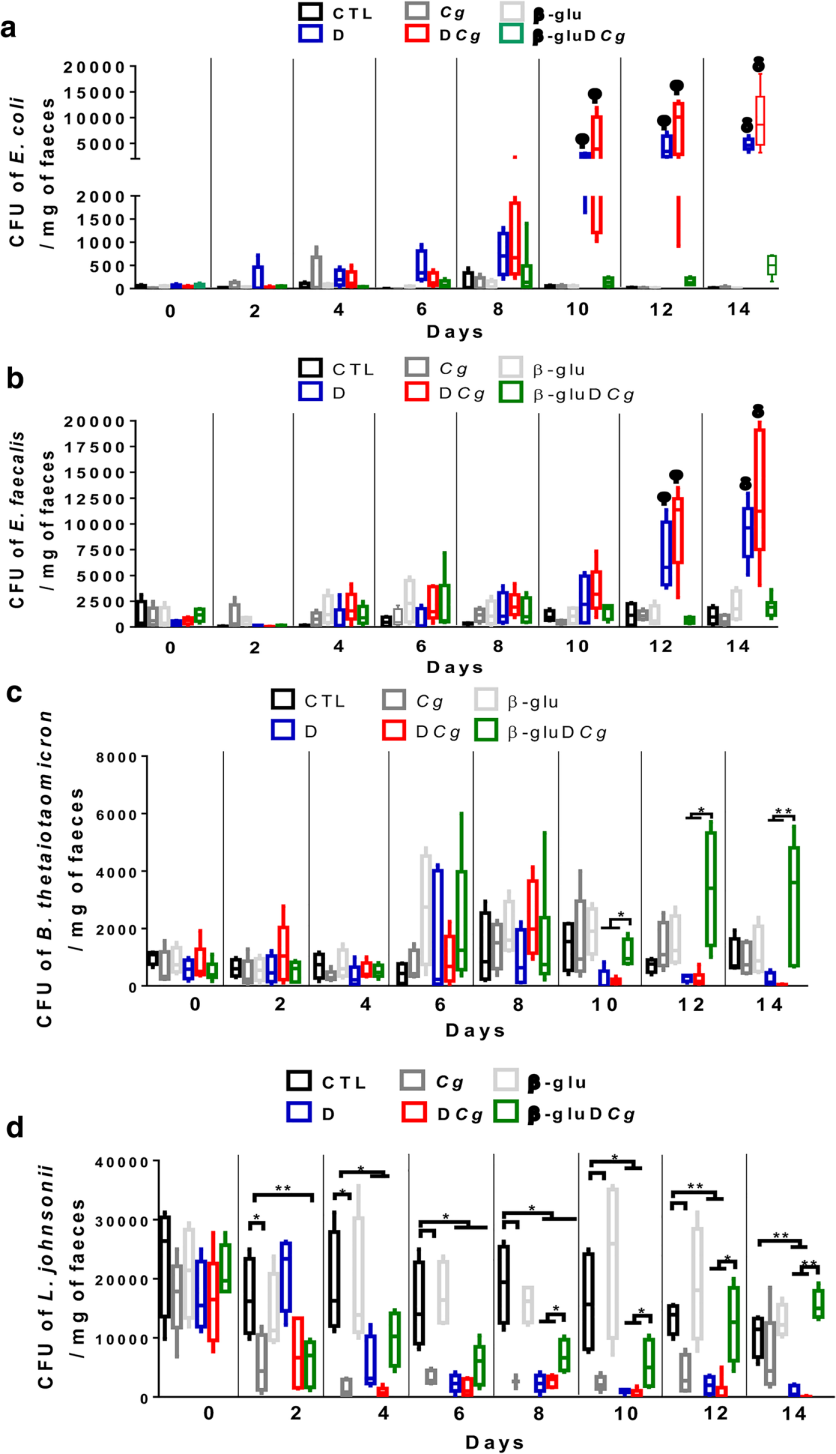
Measurement of viable fecal bacteria in DSS-induced colitis. For all experiments, stool bacteria were isolated from mice on day 0 before *C. glabrata* challenge and DSS treatment. **a**–**d** Enumeration of *E. coli*, *E. faecalis*, *B. thetaiotaomicron*, and *L. johnsonii* CFUs in stool samples (**P < 0.001, *P < 0.05)

The number of *C. glabrata* CFU was determined on day 14 in stool samples collected from each tagged mouse (Fig. 2). *C. glabrata* colonies increased significantly during the development of colitis when compared to mice given *C. glabrata* only or mice treated with β-glucans. To assess the changes in microbiota diversity during colitis development, stool samples were collected daily from each tagged mouse, and bacteria were isolated using traditional culture methods based on agar plates. The number of *E. coli* and *E. faecalis* colonies increased from day 10 to day 14 in both DSS and *C. glabrata*-DSS groups when compared to controls, *C. glabrata* or β-glucan groups, suggesting that regardless of *C. glabrata* colonization, colitis development promotes an increase in *E. coli* and *E. faecalis* populations in mice (Fig. 3).

**Fig. 4:**
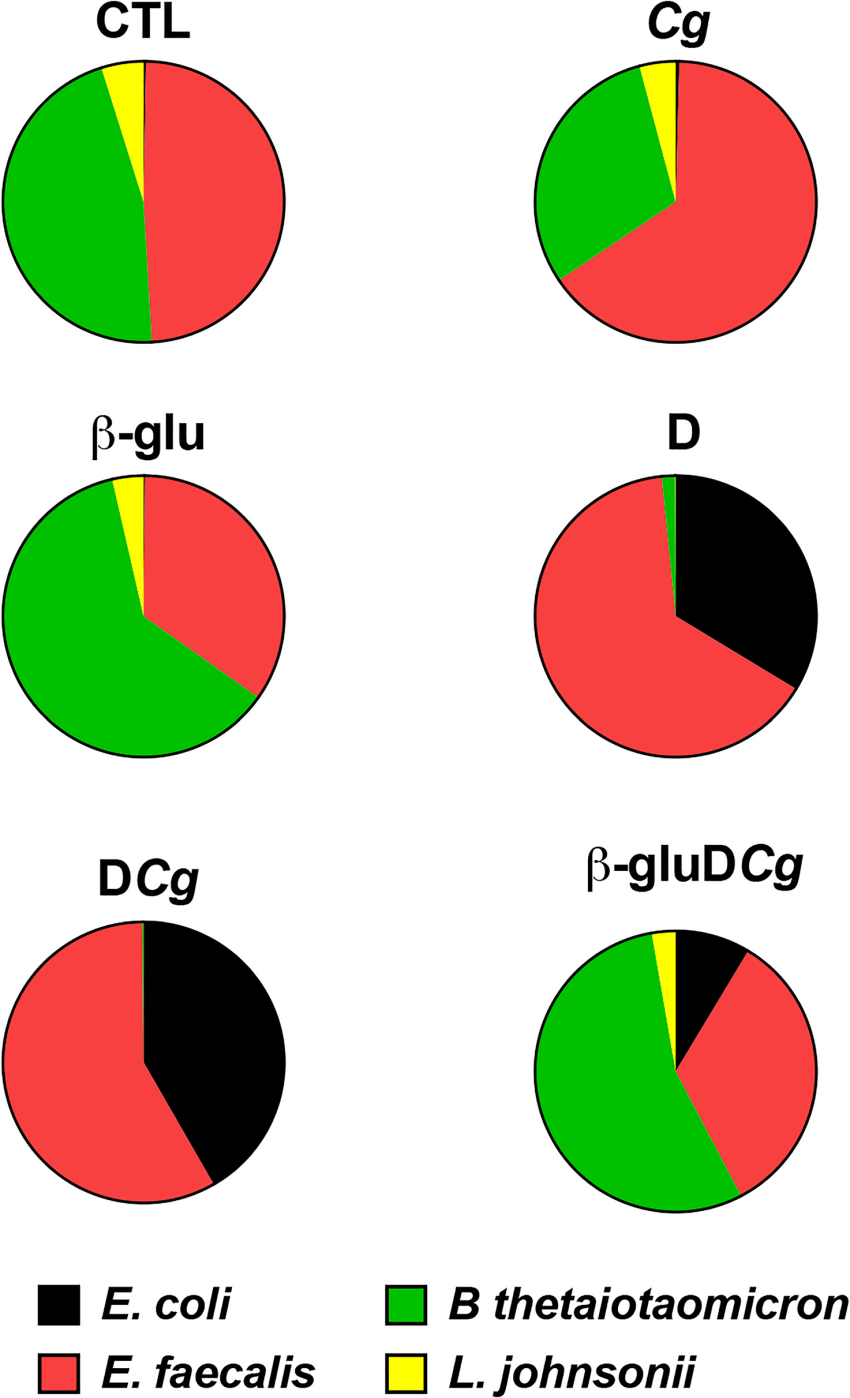
Summary of the effects of β-glucan treatment on modulation of cultivable microbiota biodiversity in *C. glabrata*-DSS treated mice. Each color corresponds to the average percentage of each analyzed species of bacteria according to fourteen days of the experiment

In terms of anaerobic bacteria, the number of *B. thetaiotaomicron* decreased significantly, starting from day 10 to day 14 in both DSS and *C. glabrata*-DSS mice, while treatment of mice with β-glucans increased the number of *B. thetaiotaomicron*. The number *of L. johnsonii* colonies was reduced significantly starting from day 2 to day 14 in both DSS and *C. glabrata*-DSS mice, but the reduction in the *L. johnsonii* population was significantly more pronounced in the *C. glabrata*-DSS group, suggesting that *C. glabrata* overgrowth has an impact on the *L. johnsonii* population during the development of colitis (Fig. 3). In contrast, treatment of mice with β-glucans significantly increased the number of *L. johnsonii* in *C. glabrata*-DSS mice (Fig. 4).

### Analysis of cytokine and receptor expression

**Fig. 5:**
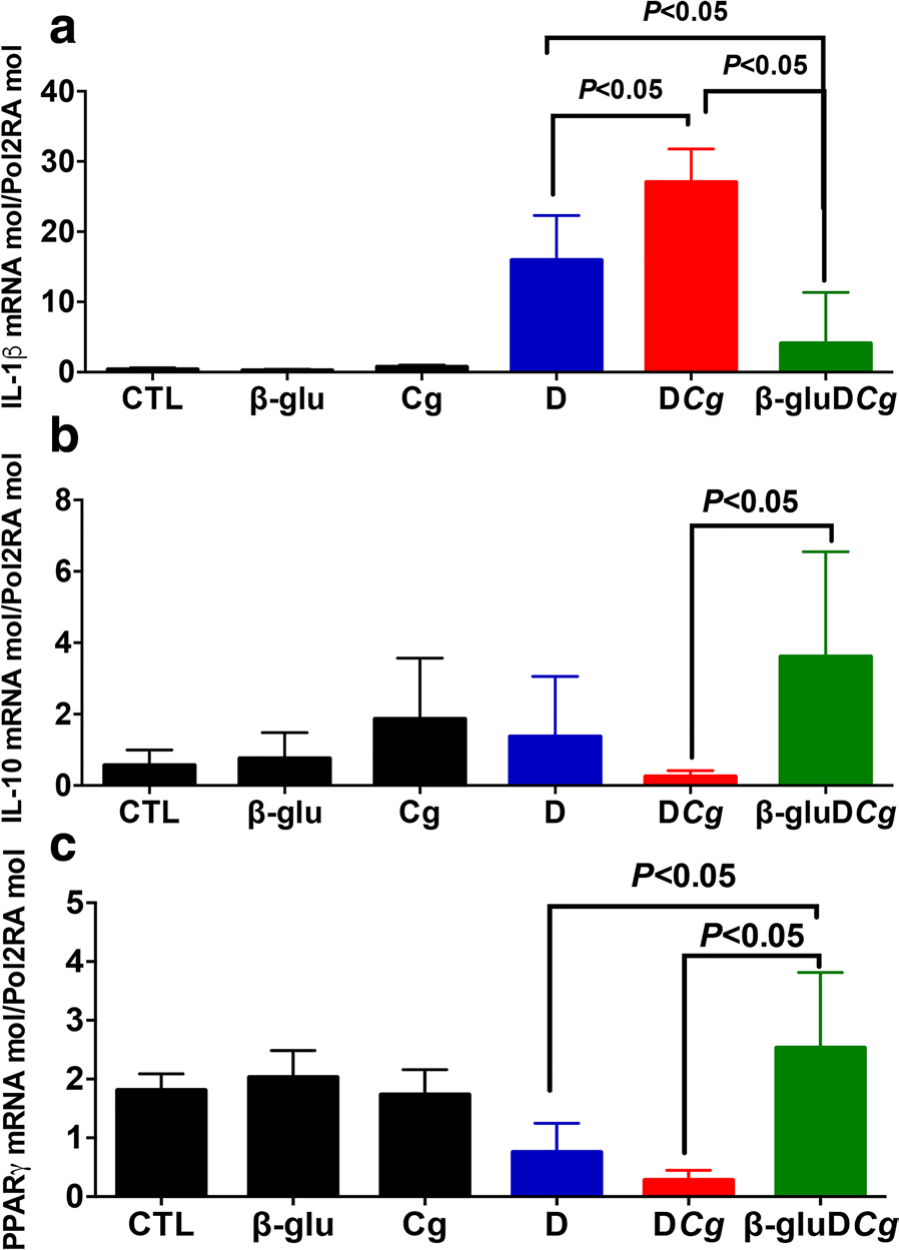
Cytokine and receptor expression in colons. **a**–**c** Relative expression levels of IL-1β, IL-10, and PPARγ mRNA in mouse colons (*P < 0.05)

Expression of IL-1β was significantly higher in the colons of *C. glabrata*-DSS mice than in DSS treated mice, whereas the expression of this cytokine was significantly lower in the colons of mice treated with β-glucans (Fig. 5). Additionally, expression of IL-10 was significantly lower in the colons of *C. glabrata*-DSS or DSS alone groups. Conversely, treatment of mice with β-glucans significantly increased the expression of IL-10 in *C. glabrata*-DSS mice. In terms of PPARγ expression, β-glucan treatment significantly increased the expression of this receptor in the colons of *C. glabrata*-DSS groups (Fig. 5).

## Discussion

IBDs are a group of chronic multifactorial disorders. Although the etiology of IBDs remains unknown, several studies have reported that the immune system, genetic susceptibility, and the environmental factors have all been implicated in its pathogenesis [1, 14, 15]. Involvement of the gut microbiota in the pathophysiology of IBDs has been highlighted [16]. Several studies have shown reduced diversity of the gut microbiota in IBD patients [4, 17]. Furthermore, patients with CD are highly colonized with *Candida* spp., in particular, *C. albicans* [18]. Consistently, in a murine model, intestinal inflammation induced chemically by DSS promotes *Candida* spp. colonization. In turn, fungal colonization exacerbates intestinal inflammation [3, 19]. The present study focused on the most representative cultivable anaerobic and aerobic bacteria that can undergo changes during intestinal inflammation [1, 20]. We focused in particular on cultivable bacteria belonging to the phyla Firmicutes, Bacteroidetes, Proteobacteria or Actinobacteria by using selective bacterial media since these bacteria are known for their possible involvement in Crohn’s disease, i.e. *E. coli* and *E. faecalis* [21].

An increase in *E. coli* and *E. faecalis* populations in mice that developed colitis was observed. These data are consistent with clinical and experimental studies, which show an increase in *E. coli* and *E. faecalis* in CD patients [4, 20]. In addition to an increase in the population of opportunistic *E. coli*, the development of colitis is characterized by high levels of reactive oxygen species (ROS) and nitrogen species, and these are considered hazardous to bacteria. However, the *E. coli* population is armed with multiple strategies to help their survival from ROS [22]. Additionally, tissue damage during colitis could serve as potential nutrients, such as ethanolamine, to encourage *E. coli* overgrowth [23, 24].

In terms of anaerobic bacteria, *B. thetaiotaomicron* and *L. johnsonii* decreased in response to intestinal inflammation, while *C. glabrata* colonization increased during the development of colitis. Interestingly, the reduction in the *L. johnsonii* population was more pronounced in the *C. glabrata*-DSS group, suggesting that *Lactobacillus* growth can antagonize colonization by *Candida* spp. [25].

The first point of contact between *C. glabrata* and its host is the fungal cell wall, whose major constituent is β-glucan. The biological activity of β-glucans is related to their molecular structure, including their solubility, their degree of branching, and their effect on the inhibition or activation of host pattern recognition receptors [13]. This study used soluble short fractions of β-glucans, purified from *C. glabrata* in a DSS-induced colitis model. Orally administered β-glucans decreased intestinal inflammation and *C. glabrata* overgrowth. This observation is consistent with our previous study, which showed that oral administration of soluble β-glucans derived from *C. albicans* reduced leukocyte infiltration into the gut mucosa and enhanced mouse survival in the DSS model [9]. Additionally, oral administration of β-glucans increased *B. thetaiotaomicron* and *L. johnsonii* populations in *C. glabrata*-DSS groups. The combination of oat β-glucans and *Lactobacillus* spp. has consistently been shown to increase the viability of these microorganisms within food matrices [26].

The anti-inflammatory activities of β-glucans from fungi have been emphasized in different studies, which have shown that soluble β-glucans can activate or block different receptors such as CD11b/CD18, dectin-1, or TLR-4 [11, 27]. Recently, short fractions of β-glucans were shown to block the activation of platelets mediated by TLR-4 expression [13]. β-glucans can initiate the innate immune response and then trigger effective immune responses including phagocytosis and cytokine production that lead to fungal elimination. It has been shown that soluble β-glucans were captured via macrophages and dendritic cells (DCs) present in Peyer’s patches and induced DC maturation via the dectin‐1 pathway [28]. In line with this observation, α-glucan induced the maturation of DCs and was dependent on TLR2 and promoted anti-inflammatory regulatory T cell responses [29].

In the present study, *C. glabrata* overgrowth reduced IL-10 expression during the development of colitis, while β-glucan treatment increased IL-10 production via PPARγ sensing. These data are consistent with the previous study, which indicates that β-glucan treatment reduces pro-inflammatory cytokine expression [9]. Mice deficient in IL-10 showed an increase in IL-1β levels that promoted uncontrolled inflammation in the intestine [30]. Additionally, inhibition of IL-1β activity with a receptor antagonist has been shown to decrease intestinal inflammation in a murine model of colitis [31].

## Conclusions

The development of colitis promotes an increase in the aerobic bacteria population, in particular *E. coli* and *E. faecalis*, but a reduction in the anaerobic bacteria population, such as *L. johnsonii*, and *B. thetaiotaomicron*. In terms of fungal cell wall components, oral administration of β-glucans to mice decreased the overgrowth of aerobic bacteria and *C. glabrata* as well as the production of inflammatory parameters. β-glucan treatment increased IL-10 production via PPARγ sensing, promoting the attenuation of colitis and *C. glabrata* elimination. We are currently analyzing the metabolites released by anaerobic bacteria during the development of colitis to determine the molecules involved in the anti-inflammatory process. In a similar way, our previous work showed an interaction between *Candida* spp., *E. coli*, and *Serratia marcescens* to form robust biofilms where lipopolysaccharide produced by *S. marcescens* and *E. coli* significantly increased fungal biofilm maturation [4].

## Methods

### *C. glabrata* strain and culture conditions

*Candida glabrata* wild-type strain (ATCC, Cg) is used. The yeast is grown in liquid YPD medium (yeast extract 1%, peptone 1%, dextrose 1%), on a rotary shaker for 18 h at 37 °C. The culture obtained is then centrifuged at 2500 rpm for 5 min and washed twice in phosphate buffer saline (PBS).

### Extraction of β-glucans from *C. glabrata*

The extraction and assay of β-glucans are carried out as described previously [9]. Briefly, *C. glabrata* cell pellet was treated twice with 200 mL of 1 M NaOH at 70 °C for 30 min. The cell pellet was then washed twice with distilled water, the supernatant was discarded and the pellet was oxidized with 100 mM NaIO4 (Sigma-Aldrich, France) at room temperature for 3 days in the dark room. After completion of the reaction, excess periodate was destroyed by adding ethylene glycol. The reduction of the pellet was performed with 1 M NaBH4 (Sigma Aldrich, France) at room temperature. The reaction was ended by decreasing the pH to 5 by the addition of acetic acid. After different times of washing with distilled water, the insoluble fraction was incubated with zymolyase 20T (0.2 mg/mL, Immuno™; ICN Biomedicals Inc.) at 37 °C for 3 h. Zymolyase was inactivated at 80 °C for 10 min. After centrifugation, the supernatant was dialyzed against distilled water. The dialyzed solution was loaded onto a Sep-Pak C18 column (Alltech) equilibrated with 0.1% trifluoroacetic acid. The evaporation of the Eluate from Sep-Pak column was performed and the resulting oligoglucosaccharides were resuspended in distilled water. Further, these oligoglucosaccharides were loaded onto a carbograph column (Alltech carbograph SPE column). Eluate from the carbograph column was the soluble β-glucan fractions. The nature and purity of the soluble β-glucan fractions are then confirmed by mass spectrometry and NMR analyses. The concentration of β-glucan fractions is measured using sulfuric acid and phenol (5%) in 96-well plates [9, 13].

### Animals

Ten-to-12-week-old, female C57BL/6 mice are used (Charles River Laboratories, France). Mice are allocated to one of three experimental groups or three control groups. A group of healthy mice is used as controls (CTL, 5 mice). A second group of mice receive oral gavage with *C. glabrata* without any other treatment (Cg, 5 mice). A third group are treated with β-glucan fractions (β-glu, 5 mice). A fourth group are treated with DSS (D, 10 mice). A fifth group are treated with DSS and oral gavage with *C. glabrata* (DCg, 12 mice). A sixth group are given DSS and oral gavage with *C. glabrata* and treated with β-glucans (β-gluDCg, 6 mice).

All animal experiments were approved by the subcommittee for Research Animal Care, Regional Hospital Center, Lille, France (00550.05), and were in accordance with institutional (86/609/CEE) and European guidelines for the care and use of laboratory animals.

### Inoculum preparation and induction of colitis

The mice are given 200 µL PBS containing 5 × 10^7^ live *C. glabrata* cells on day 1 by oral gavage. From day 1 to day 14, mice are also treated with 2% DSS (36–50 kDa; MP Biomedicals, LLC, Germany) in drinking water in order to induce colitis. For β-glucan treatment, mice are given β-glucans purified from *C. glabrata* (3 mg per mouse) orally and daily for 5 days, starting on day 1. The presence of *C. glabrata* in the gut is determined by measuring the number of colonies in feces (approximately 0.1 g/sample) collected from each animal at day 14. Fecal samples are suspended in 1 mL PBS, and samples are then cultured on Candi-Select medium (Bio-Rad Laboratories, France). The results are expressed as CFU/mg of feces [32].

### Isolation of bacteria

Serial dilutions of fecal samples collected from the mice are performed daily. The samples are cultured on non-selective bacterial media (AC agar) focusing on the most representative cultivable anaerobic and aerobic bacteria that can undergo changes during intestinal inflammation. Isolation of aerobic bacteria and anaerobic bacteria is carried out as described previously [33]. Identification of bacteria isolated on selective media is performed by MALDI TOF mass spectrometry [33].

### Assessment of clinical and histologic scores

The body weight of the mice is recorded daily. Data are expressed as mean percent change from initial body weight. Clinical scores ranging from 0 to 12 and histologic scores ranging from 0 to 6 are assessed as described previously [19, 33].

### Real-time mRNA quantification of cytokines and receptor expression

Nucleospin RNA kit is used to extract total RNA from mouse colons (Macherey–Nagel). RNA is measured by spectrophotometry (Nanodrop; Nyxor Biotech, France). mRNA reverse transcription is performed from 1 µg total RNA using a high capacity cDNA-RT kit (Applied Biosystems) in a final volume of 20 µL [34, 35]. cDNA synthesis is carried out by PCR in the one-step system (Applied Biosystems) using Fast SYBR green (Applied Biosystems). SYBR green dye intensity is assessed using one-step software [35]. The reference gene used to normalize the results is POLR2A47 [33].

### Statistical analysis

All data are expressed as the mean ± standard deviation for each experimental group. Pairs of groups are compared using the Mann–Whitney U test. Data are considered to be statistically significant when the *P* value is as follows: P < 0.05; P < 0.01; P < 0.001. All statistical analyses are performed using GraphPad Prism 4.0 and XLSTAT.
